# Effect of Bi_2_O_3_–CuO Flux on the Microstructure, Soft Magnetic Properties, and Gyromagnetic Properties of NiCuZn Ferrites for LTCC Devices

**DOI:** 10.3390/mi15020215

**Published:** 2024-01-31

**Authors:** Xiaoling Lu, Lei Zhang

**Affiliations:** 1 State Key Laboratory of Dynamic Measurement Technology, North University of China, Taiyuan 030051, China; 18428381577@163.com; 2 Key Laboratory of Micro/Nano Devices and Systems, Ministry of Education, North University of China, Taiyuan 030051, China

**Keywords:** NiCuZn ferrite, Bi_2_O_3_–CuO doping, gyromagnetic properties, soft magnetic properties

## Abstract

In this work, the electromagnetic properties of Ni_0.22_Cu_0.31_Zn_0.47_Fe_2_O_4_ (NiCuZn) ferrites doped with 0.3 wt% Bi_2_O_3_ + *x*CuO flux (*x* = 0.2, 0.4, 0.6, and 0.8 wt%) were studied. Doping resulted in a reduction in the sintering temperature to 900 °C. The doped ferrites were synthesized via the solid-state method. XRD patterns revealed that the prepared ferrites had a cubic spinel structure; thus, a moderate addition of flux did not change the crystal structure. The SEM images, as well as the density and grain size distribution of the samples, showed that the NiCuZn ferrites had densified, homogenized, and contained fully grown grains for *x* = 0.6 wt%. The sample exhibited good soft magnetic properties, with μ′ reaching the maximum value of 245.4 for *x* = 0.6 wt% and ε′, *M_s_*, and *H_c_* reaching the maximum values of 23.1, 28.06 emu/g, and 45.86 Oe for *x* = 0.8 wt%, respectively. Furthermore, the ferrites exhibited good gyromagnetic properties, with *4πM_s_* reaching the maximum value of 1744 Gauss for *x* = 0.8 wt% and ΔH reaching the minimum value of 228 Oe for *x* = 0.6 wt%. NiCuZn ferrites were successfully sintered at a lower temperature (900 °C) by adding Bi_2_O_3_–CuO flux through LTCC technology and exhibited good soft magnetic properties and gyromagnetic properties. We envisage that these ferrites could be used in multilayer devices.

## 1. Introduction

Nowadays, the rapid development of the electronic information industry, such as the Internet of Things, has accelerated the miniaturization, integration, and diversification of various electronic devices. Low-temperature co-fired ceramic (LTCC) technology can be utilized to further improve the miniaturization and integration of electronic devices. The increasing demand for the miniaturization of components has stimulated extensive research into LTCC technology. However, since LTCC technology requires the ceramics to be co-fired with a Ag electrode, the sintering temperature of ferrites needs to be lowered to below 961 °C (the Ag melting point). Among the many existing ferrite ceramics, NiCuZn ferrites have excellent properties and have thus received widespread attention [1,2,3,4]. NiCuZn ferrites cannot be used in the field of three-dimensional integrated devices due to their high sintering temperatures (above 1400 °C) [5,6]. Therefore, it is necessary to develop new NiCuZn ferrite materials with excellent microwave performance and a lower sintering temperature. To this end, doping these ferrites using LTCC technology is a promising strategy.

According to previous reports, in order to meet the various developmental requirements of different components, the most common approach to tuning and improving the microstructure and magnetic properties of low-temperature-sintered NiCuZn ferrites relies on doping or substitution [7,8,9]. Bi_2_O_3_ is the most common oxide used to dope low-temperature-sintered NiCuZn ferrites. Patil et al. studied the optical and magnetic properties of Nb_2_O_5_-doped NiCuZn ferrites. They found that the addition of Nb_2_O_5_ reduced the bandgap energy, saturation magnetization, and coercivity of the sample [9]. Almessiere et al. studied the electrical, magnetic, and microwave properties of Dy-substituted NiCuZn ferrites. They showed that increasing the Dy content led to a decrease in the average crystallite size, and the bandgap energy was inferred to be between 1.83 and 1.86 eV. Furthermore, they discussed the microwave characteristics in the frequency range of 1–20 GHz and observed a strong electromagnetic absorption in the frequency range of 1.6–2.7 GHz [10]. Ji et al. studied the electromagnetic properties of Bi_2_O_3_-substituted NiCuZn ferrites prepared via LTCC. They reported that the electromagnetic properties of NiCuZn ferrites were enhanced upon addition of 0.30 wt% Bi_2_O_3_; they obtained a high real part of the permeability (∼937.6 @1 MHz), a high saturation magnetization (∼60.353 emu/g), a low coercivity (∼0.265 kA/m), and an excellent dielectric constant (∼14.71 @1 MHz) [11]. Low-melting-point metal oxides, such as CuO [3,12], Mn_2_O_3_ [13], V_2_O_5_ [14], and Nb_2_O_5_ [15], have also been added to NiCuZn ferrites to effectively reduce their sintering temperature while retaining a good performance. However, the soft magnetic properties and gyromagnetic properties of NiCuZn are also important in determining their device applications.

In this work, we used a solid-state reaction method and low-temperature sintering to dope NiCuZn ferrite samples with a Bi_2_O_3_–CuO flux. The addition of Bi_2_O_3_–CuO can not only tailor the performance of the sample but also reduce its sintering temperature. We added 0.3 wt% Bi_2_O_3_ and variable amounts of CuO (0.2, 0.4, 0.6, and 0.8 wt%) to the ferrites, and the microstructure, soft magnetic properties, and gyromagnetic properties of the doped ferrites were systematically studied. Selecting an appropriate composition of the NiCuZn ferrite is also important in achieving a good performance. NiCuZn ferrites have the spinel structure with a cubic close-packed lattice, and their molecular formula is AB_2_O_4_, where A represents a divalent metal and B represents a trivalent metal, usually the Fe^3+^ ion. The A and B cations in both sites can be partially substituted by different metal cations with a suitable ionic radius to obtain new materials with novel properties. According to one of our previous works [16], we selected the following composition for this study: Ni_0.22_Cu_0.31_Zn_0.47_Fe_2_O_4_. Eventually, an alternative NiCuZn ferrite ceramic with excellent performance was successfully synthesized. This ferrite ceramic has considerable potential for use in microwave circulators and filters in the LTCC field.

## 2. Materials and Methods

The Ni_0.22_Cu_0.31_Zn_0.47_Fe_2_O_4_ (here denoted as NiCuZn) samples were synthesized via a two-step solid-state sintering method. The powders (Fe_2_O_3_, ZnO, NiO, and CuO; purity ≥ 99%; Kelong, Chengdu, China) were weighed in stoichiometric amounts. Then, deionized water was added to the powders, and the powders were mixed in a ball mill (Nanjing Machine Factory, Nanjing, China) with nylon balls (the balls were 10 or 6 nm in diameter) at a running speed of 220 rpm for 24 h. The spinel-phase NiCuZn ferrites were prepared using the calcination method at 800 °C for 3 h at a heating rate of 2 °C/min. The calcined powders were mixed with 0.3 wt% Bi_2_O_3_ and different amounts of CuO (*x* = 0.2, 0.4, 0.6, and 0.8 wt%) and ball milled again at 220 rpm for 24 h. These mixtures were pressed into samples with the polyvinyl alcohol binder (10 wt%) under a pressure of 12 MPa. The prepared samples were in the form of cylinders, rings, and powder. The powder was used for the phase analysis. The cylinder- and ring-shaped samples were used to determine the permittivity and permeability of the ferrites. Finally, the samples were sintered in atmospheric pressure at 900 °C for 12 h and then cooled naturally.

The phase of the NiCuZn ferrites was identified via X-ray diffraction (XRD) (D/max 2400, Rigaku, Tokyo, Japan) with Cu Kα radiation. Scanning electron microscopy (SEM, JEOL-JSM-6490, Japan Electron Optics Laboratory, Tokyo, Japan) was used to investigate the morphology and microstructure of the prepared samples. The density of the samples was measured using Archimedes’ method and was calculated according to the following expression:

(1) $$\rho =\frac{m_{0}\rho_{0}}{m_{1}-m_{2}},$$

where $\rho_{0}$ is the density of distilled water, $m_{0}$ is the mass of the sample in air, $m_{1}$ is the mass of the sample after the gaps are filled with distilled water, and $m_{2}$ is the mass of the sample immersed in water. The magnetic hysteresis loops were measured using a vibrating sample magnetometer (VSM) (BHV-525, Riken Denshi, Tokyo, Japan) with an applied magnetic field up to 2500 Oe. The average grain size was determined based on the SEM images through a linear intercept method. The complex permeability was measured using an impedance analyzer (E4991B, Agilent, Santa Rosa, CA, USA) at a frequency varying from 1 MHz to 1 GHz. The ferromagnetic resonance (FMR) line width (ΔH) of the samples required preparation of spherical samples with a diameter of about 1.0 mm, and was measured using the TE106 cavity perturbation method at ~9.55 GHz. All measurements were carried out at room temperature.

## 3. Results

### 3.1. Phase and Microstructure

**Figure 1:**
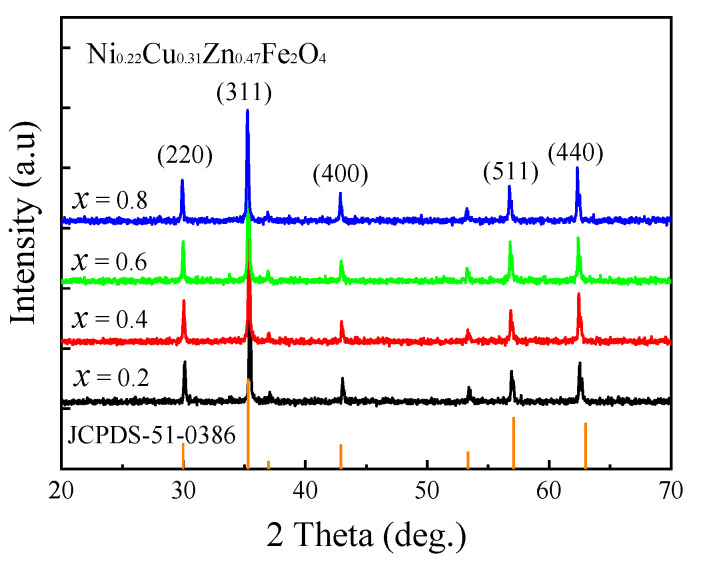
XRD patterns of the NiCuZn samples with 0.3 wt% Bi_2_O_3_ + *x*CuO addition (*x* = 0.2, 0.4, 0.6, and 0.8 wt%).

Figure 1 shows the XRD patterns of the Ni_0.22_Cu_0.31_Zn_0.47_Fe_2_O_4_ ferrites doped with 0.3 wt% Bi_2_O_3_ + *x*CuO (*x* = 0.2, 0.4, 0.6, and 0.8 wt%). The diffraction patterns revealed that all the ferrites exhibited the cubic spinel single-phase structure (JCPDS card No. 51-0386); the XRD peaks corresponded to the (220), (311), (400), (511), and (440) crystal planes of this structure, as shown in Figure 1. The XRD patterns demonstrated that the low-temperature sintering (900 °C) of the doped NiCuZn samples was successful, and the addition of a small quantity of Bi_2_O_3_–CuO had no influence on the formation of the NiCuZn polycrystalline spinel structure.

**Figure 2:**
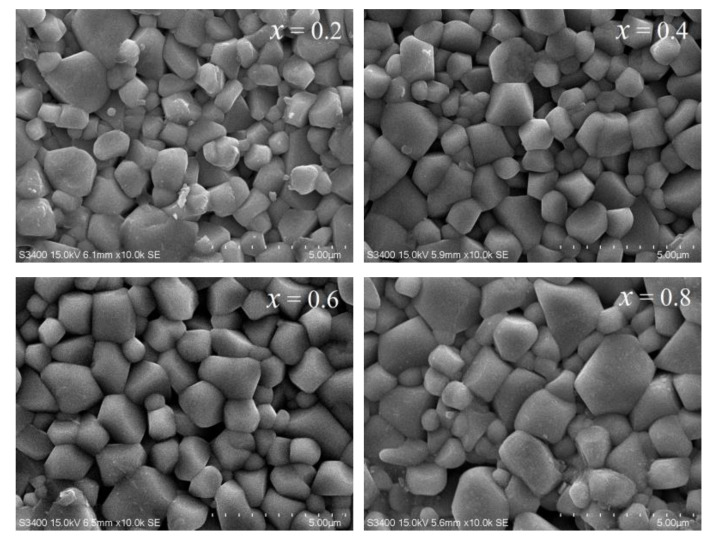
SEM images of the NiCuZn samples with 0.3 wt% Bi_2_O_3_ + *x*CuO addition (*x* = 0.2, 0.4, 0.6, and 0.8 wt%).

Figure 2 shows the SEM images of the NiCuZn samples with different Bi_2_O_3_–CuO contents at ×10K magnification. The crystal grains and grain boundaries could be clearly observed in all samples. These SEM results indicate that the addition of 0.3 wt% Bi_2_O_3_ and various amounts of CuO was beneficial for reducing the sintering temperature of NiCuZn, so that the polycrystalline phase could be successfully synthesized at a sintering temperature of 900 °C. As the CuO content increased, the particle size and pores changed. Specifically, for *x* = 0.2 wt% (the Bi_2_O_3_ amount was constant at 0.3 wt%), the microstructure of the sample was not dense, with a small number of small grains and numerous pores. As x increased to 0.6 wt%, the grain size gradually became uniform, the density of the sample increased, and the number of pores decreased. As x increased further to 0.8 wt%, a structure combining small and large grains appeared; furthermore, the grains were extremely large and not uniform in size. This phenomenon was related to the CuO flux. The increase in CuO content can effectively promote the densification of the sample, but as the CuO content exceeds a certain threshold, a typical bimodal heterogeneous structure appears; this is similar to what happens when over-doping a material. Therefore, when Bi_2_O_3_–CuO was added, a solution–reprecipitation process occurred. At this point, the activation energy required for grain growth decreased, and the grain size and density increased accordingly. Therefore, incorporating an appropriate amount of Bi_2_O_3_–CuO into NiCuZn ferrites can reduce the sintering temperature and improve the microstructure of the sample, which in turn affects the magnetic properties of the sample.

**Figure 3:**
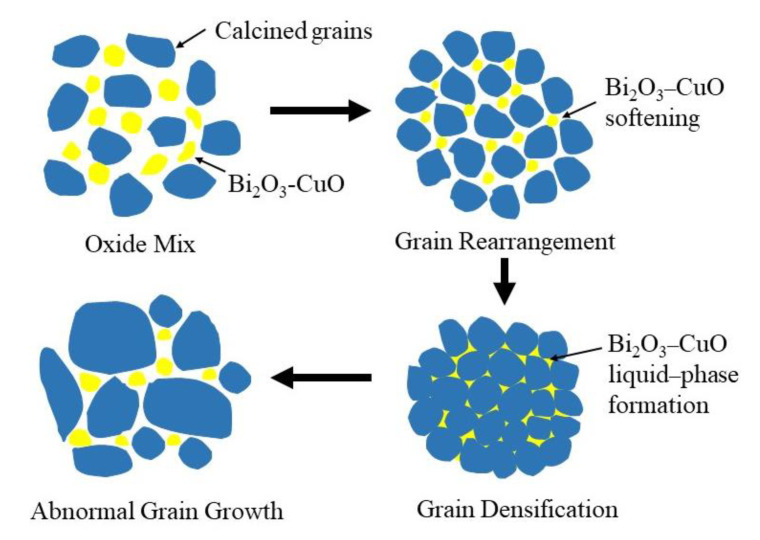
Schematic of the low-temperature sintering mechanism of the NiCuZn samples.

Figure 3 shows the schematic of the low-temperature sintering mechanism of the sample with the addition of the Bi_2_O_3_–CuO flux. When the NiCuZn ferrite was sintered at a sintering temperature greater than 800 °C, the Bi_2_O_3_–CuO flux started to soften, the crystal grains started to rearrange, and the pores started to be filled. As the sintering temperature continued to increase, the grains gradually started to grow because the capillary force generated by the liquid phase formed by Bi_2_O_3_–CuO at the grain boundaries promoted grain growth, causing the grains to increase gradually in size. As the sintering temperature was further increased to 900 °C, that is, the final stage of the solid-state sintering process, the grains had sufficient energy to grow fully, and they were homogenized and densified; this was how the fully reacted NiCuZn ferrite was finally obtained via low-temperature sintering. The Bi–O and Cu–O ionic bonds in the sample absorbed energy, broke, and ionized; therefore, the addition of an excessive Bi_2_O_3_–CuO flux amount led to an excessive number of free Cu^2+^ and Bi^3+^ ions, which resulted in abnormal grain growth.

**Figure 4:**
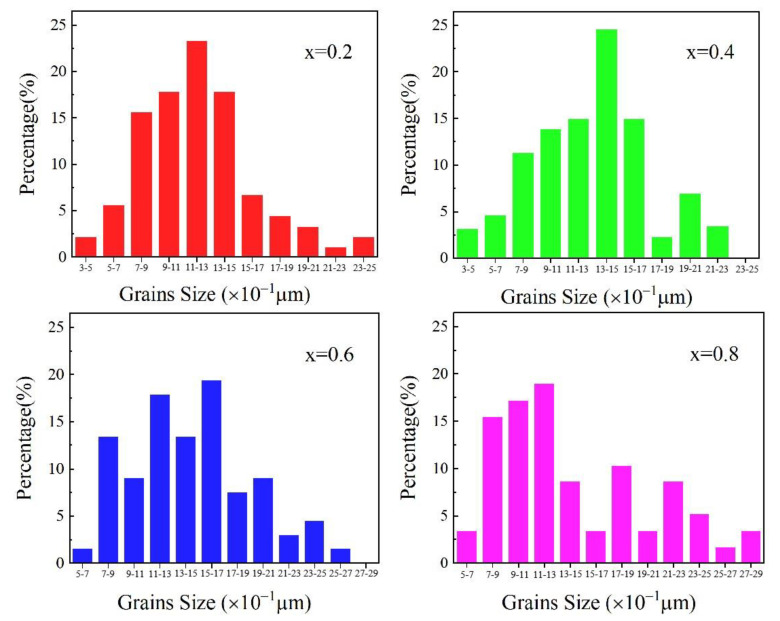
Grain size distributions of the NiCuZn samples with 0.3 wt% Bi_2_O_3_ + *x*CuO addition (*x* = 0.2, 0.4, 0.6, and 0.8 wt%).

Figure 4 shows the grain size distribution of the samples with different Bi_2_O_3_–CuO flux contents. As shown in Figure 4, as the Bi_2_O_3_–CuO content increased, the average grain size gradually increased, and more large-sized grains appeared. For *x* = 0.2 wt%, approximately 86.8% of the grains had sizes in the range of 0.5–1.7 μm. For *x* = 0.6 wt%, around 89.6% of the grains have sizes in the range of 0.7–2.1 μm. It can be seen from the histograms that there were no extremely small or abnormally large grains. This was mainly due to the fact that at this sintering temperature, the addition of an optimal Bi_2_O_3_–CuO amount provided a suitable activation energy, enhanced the compactness of the sample, effectively suppressed the growth of abnormal grains, and finally resulted in the formation of a uniform and dense microstructure with a narrow grain-size distribution. For *x* = 0.8 wt%, approximately 88.2% of the grains had sizes in the range of 0.7–2.5 μm, and 3.4% of the grains were abnormally large (with sizes in the range of 2.7–2.9 μm); this was related to the excessive number of Cu^2+^ and Bi^3+^ ions that were generated at such a high x value. The obtained histograms are in agreement with the above analysis.

**Figure 5:**
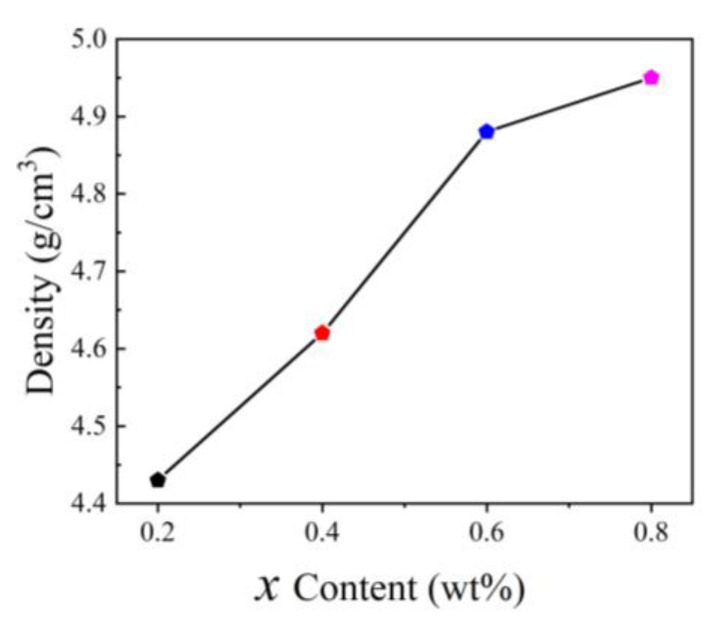
Bulk density of the NiCuZn samples with 0.3 wt% Bi_2_O_3_ + *x*CuO addition (*x* = 0.2, 0.4, 0.6, and 0.8 wt%).

Figure 5 shows the bulk density of the NiCuZn samples. The bulk density was measured via Archimedes’ method at room temperature using distilled water as the buoyancy liquid. The values of the bulk density were 4.43, 4.62, 4.88, and 4.95 g/cm^3^ for *x* = 0.2, 0.4, 0.6, and 0.8 wt%, respectively. The bulk density increased monotonically with increasing Bi_2_O_3_–CuO content and was thus consistent with the trend inferred from the SEM images. Porosity was also a key factor affecting the magnetic and dielectric properties of samples. Porosity and bulk density are closely related and there is an inverse relationship between them. It can be expressed by the following formula: Porosity = 1 − bulk density/theoretical density. The calculated data are shown in Table 1. Fitting results for the XRD patterns were accomplished using the Jade 6.0 software, and the theoretical density was calculated, and is given in Table 1. It can be seen that the porosity first decreased and then increased, which also corresponded to the microstructure of the SEM image. The density of a sample is related to its magnetic, dielectric, and gyromagnetic properties. Analysis of these properties is described in the following section.

### 3.2. Soft Magnetic Properties

**Figure 6:**
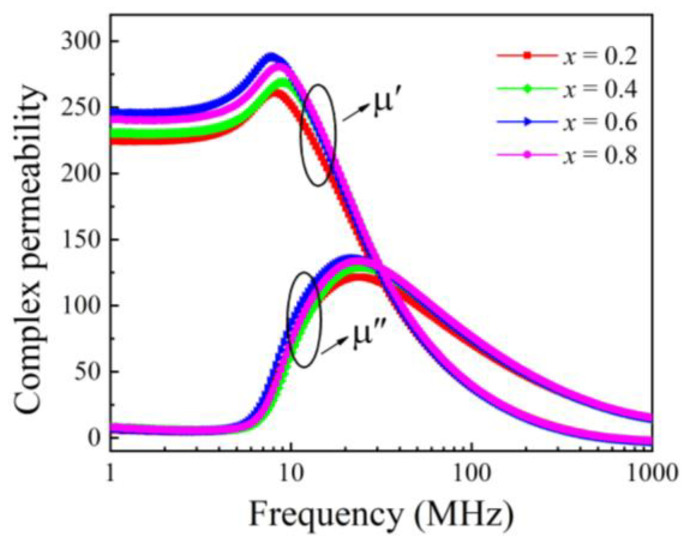
Complex permeability of the NiCuZn samples with 0.3 wt% Bi_2_O_3_ + *x*CuO addition (*x* = 0.2, 0.4, 0.6, and 0.8 wt%) as a function of frequency from 1 MHz to 1 GHz.

Figure 6 shows the variations in the complex permeability spectra (μ′ and μ″) of the samples with different Bi_2_O_3_–CuO concentrations in the frequency range of 1 MHz–1 GHz. It can be seen from the figure that with the increase in the Bi_2_O_3_–CuO flux content, the magnetic permeability (μ′ @1 MHz) first increased and then decreased, while μ″ changed to a smaller extent. Interestingly, a lower value of μ″ endows the sample with a better quality factor (Q). The μ′ values at 1 MHz were 224.2, 230.5, 245.4, and 240.5 for the four samples, respectively. The permeability increased significantly and peaked at *x* = 0.6 (μ′ = 245.4), mainly due to the increase in the sample density and composition. The magnetic permeability of ferrites is mainly determined by their composition, microstructure, porosity, and density [12]. The porosity causes the magnetic permeability of the sample to not change linearly with the doping amount, improving the increase in the magnetic permeability. In addition to the changes in porosity and density caused by the experimental process, there are many factors that affect the magnetic permeability of NiCuZn ferrite. According to the literature, the key factors are as follows: (i) the saturation magnetization *M_s_* of the NiCuZn ferrites; (ii) the magnetocrystalline anisotropy constant K1 and the hysteresis stretching coefficient λ0; and (iii) the microstructure (pore and grain sizes). The relationship between the initial permeability, saturation magnetization, and grain size can be expressed as:

(2) $$\mu_{i}\approx \frac{M_{s}^{2}D}{\sqrt{K_{1}}},$$

where *μ_i_* is the magnetic permeability and *D* is the average grain size. The *M_s_* of the samples is closely related to their composition. The Ni_0.22_Cu_0.31_Zn_0.47_Fe_2_O_4_ composition used in this work had a better saturation magnetization than other NiCuZn compositions [17,18]. Indeed, the *M_s_* value of NiCuZn ferrites is unlikely to vary significantly, and increasing *M_s_* results in a higher *K*_1_, as shown by the following equation:

(3) $$K_{1}=\frac{M_{s}H_{c}}{0.96},$$

where *H_c_* is the coercive field. By substituting Equation (3) into Equation (2), it can be seen that the change in magnetic permeability is mainly related to the saturation magnetization and the average grain size. With the increase in the Bi_2_O_3_–CuO content, due to the liquid-phase sintering mechanism, the grain growth in the NiCuZn ferrites is promoted, the grains increase in size until they are fully grown, and the uniformity and compactness of the ferrites increase. The best low-temperature-sintered NiCuZn ferrite was obtained for *x* = 0.6 wt%. For this Bi_2_O_3_–CuO content, the magnetic permeability was the highest (245.4). For *x* = 0.8 wt%, the permeability was reduced due to the occurrence of abnormal grain growth. Therefore, by adding Bi_2_O_3_–CuO to NiCuZn ferrites, their microstructure can be tailored, and the magnetic properties can be correspondingly tuned.

**Figure 7:**
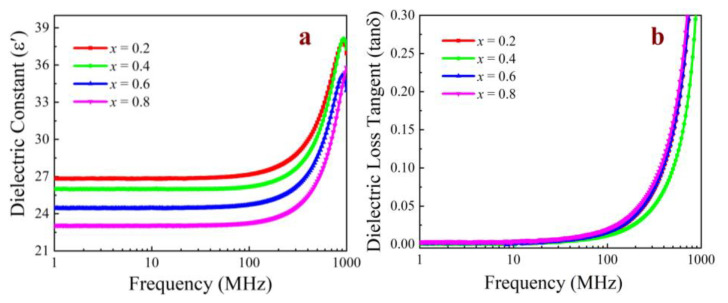
*ε*′ and tan*δ* of the NiCuZn samples with 0.3 wt% Bi_2_O_3_ + *x*CuO addition (*x* = 0.2, 0.4, 0.6, and 0.8 wt%) as a function of frequency from 1 MHz to 1 GHz. (**a**) Dielectric constant *ε*′ of NiCuZn, (**b**) Dielectric loss tangent.

Figure 7 shows the complex permittivity of the NiCuZn ferrites as a function of frequency between 1 MHz and 1 GHz. Figure 7a shows the measured dielectric constant *ε*′, and Figure 7b shows the measured dielectric loss tangent (tan*δ* = *ε*″/*ε*′). As shown in the figure, the values of *ε*′ (@1 MHz) for *x* = 0.2, 0.4, 0.6, and 0.8 wt% were 26.8, 25.9, 24.4, and 23.1, respectively. The dielectric constant of the sample did not change linearly with the doping amount because, in addition to the change in electronic polarization and microstructural changes caused by the doping amount, the porosity also affects the dielectric constant of the sample. This resulted in a difference between the theoretically predicted value of the dielectric constant of the sample and the experimental value, and experiments were necessary. This dependence of *ε*′ on the CuO content may have been related to electronic polarization and microstructural changes. The value of *ε*′ (@1 MHz) decreased with increasing Bi_2_O_3_–CuO content, which was due to the fact that the Bi^3+^/Cu^2+^ ions increased the local charge and reduced the dielectric constant. The Bi^3+^/Cu^2+^ ions present in the sample had a strong conductivity and a low dielectric constant. The permittivity remained stable over a wide frequency range, which is a typical dielectric behavior of NiCuZn ferrites [16]. According to previous studies, NiCuZn ferrites are mainly characterized by four polarization mechanisms, namely electronic polarization (*α*_e_, which occurs at 10^15^ Hz), ionic polarization (*α*_a_, which occurs at 10^10^–10^13^ Hz), dipolar polarization (*α*_o_, which occurs at 10^3^–10^6^ Hz), and interfacial polarization (*α*_i_, which occurs below 10^3^ Hz). These different polarization mechanisms contribute differently to the overall polarization of the ferrites at different frequencies. However, as the NiCuZn ferrites were prepared via the traditional solid-state reaction method, their dielectric behavior was affected by many other factors in addition to the polarization, including grain boundary defects, changes in the free charges, and distortion effects. The dielectric loss had a similar behavior to that of *ε*′. This may be correlated to the fact that domain wall motion has droop values, and hence the losses were lower. The dielectric loss tangent was small and ranged from 0.5 × 10^−3^ to 3 × 10^−3^. The presence of Cu^2+^ ions in the samples is one of the reasons for the small dielectric loss tangent across the investigated frequency band. tan*δ* can be expressed as:

(4) $$\tan\delta =(1-P)\tan\delta_{0}+CP^{n},$$

where tan*δ*_0_ is the dielectric loss of a densely structured material, *P* is the porosity, and *C* is a constant. Therefore, obtaining a suitable NiCuZn ferrite structure with a low number of pores at an appropriate CuO content is important for reducing the dielectric loss. The dielectric loss tangent is a key evaluation parameter when a material is used in an electronic device, as large dielectric losses result in electrical energy consumption and cause the device to heat up. This heating can damage the insulation and even affect the normal operation of the device.

**Figure 8:**
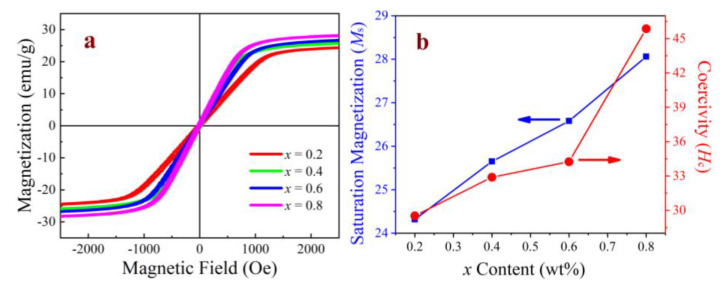
Magnetic properties of the NiCuZn samples with 0.3 wt% Bi_2_O_3_ + *x*CuO addition (*x* = 0.2, 0.4, 0.6, and 0.8 wt%). (**a**) *M*–*H* curves measured up to 2.5 kOe and (**b**) *M_s_* and *H_c_* for the different samples.

Figure 8a shows the magnetic hysteresis loops (*M*–*H*) measured up to 2.5 kOe at room temperature. All NiCuZn samples showed a typical soft magnetic behavior and a low coercivity under an external magnetic field. The specific values of the *M_s_* and *H_c_* of the ferrites with different Bi_2_O_3_–CuO contents were calculated and are displayed in Figure 8b. The results show that *M_s_* and *H_c_* gradually increased with increasing *x*. For *x* = 0.2, 0.4, 0.6, and 0.8 wt%, the *M_s_* values were 24.32, 25.65, 26.58, and 28.06 emu/g, respectively, and the *H_c_* values were 29.52, 32.89, 34.24, and 45.86 Oe, respectively. The variation in *M_s_* with CuO content can be attributed to the modification of the microstructure of the ferrites due to the incorporation of the magnetic ions (Cu^2+^). It has been reported that a high density and a uniform grain size are beneficial for obtaining ferrites with a high *M_s_* value. As discussed above, the increase in the Cu^2+^ ion content led to an increase in the ferrite density and grain size, which in turn increased the *M_s_* value. With the introduction of Cu^2+^ ions, the defects of the sample were increased, which affected the pinning and increased the resistance to domain wall displacement, thereby increasing the *H_c_*.

### 3.3. Gyromagnetic Properties

**Figure 9:**
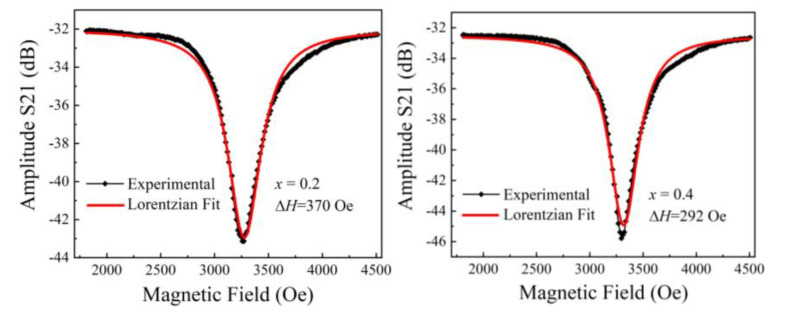

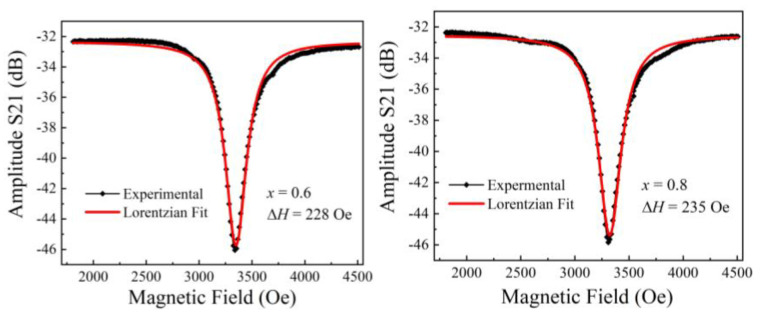
FMR curves of the NiCuZn samples with 0.3 wt% Bi_2_O_3_ + *x*CuO addition (*x* = 0.2, 0.4, 0.6, and 0.8 wt%).

Figure 9 shows the FMR linewidth (Δ*H*) of the NiCuZn samples with various amounts of added Bi_2_O_3_–CuO and the corresponding fittings using a Lorentzian function. The experimental data were fitted well using the following Lorentz distribution:

(5) $$y=y_{0}+\left(2\times A/\pi \right)\times \left\{w/\left[4\times {\left(x-x_{c}\right)}^{2}+w^{2}\right]\right\},$$

where *A* is the area between the curve baseline and the curve and *w* is the full width at half maximum; the other fitting parameters are listed in Table 2. The *R*^2^ values of the parameters derived from the fitting were all greater than 0.987, demonstrating the validity of using a Lorentz distribution to fit the data. The Lorentzian curve fits the data better when the FMR linewidth is smaller.

**Figure 10:**
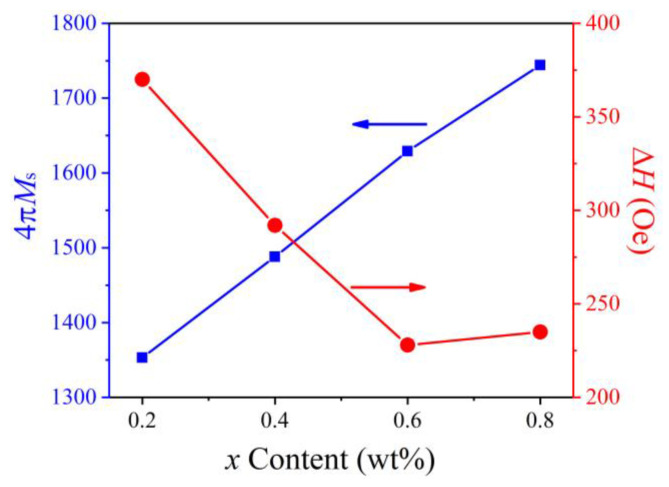
Values of 4π*M*_s_ and Δ*H* of the NiCuZn samples with 0.3 wt% Bi_2_O_3_ + *x*CuO addition (*x* = 0.2, 0.4, 0.6, and 0.8 wt%).

Figure 10 shows the 4π*M_s_* and Δ*H* of the NiCuZn ferrites as a function of the Bi_2_O_3_–CuO content. The value of 4π*M*_s_ was determined by the saturation magnetization and density of the sample. The results show that as the Bi_2_O_3_–CuO content increased, 4π*M*_s_ increased monotonously, while Δ*H* first decreased and then increased. For *x* = 0.08 wt%, 4π*M_s_* reached the maximum value of 1744 Gauss. For *x* = 0.06 wt%, Δ*H* reached the minimum value of 228 Oe. An appropriate amount of Bi_2_O_3_–CuO promotes grain growth in the NiCuZn ferrites, which increases the *M_s_*, density, and average grain size, resulting in an increase in 4π*M_s_*. For *x* = 0.2, 0.4, 0.6, and 0.8 wt%, the values of 4π*M_s_* were 1353, 1488, 1629, and 1744 Gauss, respectively, and the Δ*H* values were 370, 292, 228, and 235 Oe @9.3 GHz, respectively. Both 4π*M_s_* and Δ*H* are important gyromagnetic parameters of microwave ferrites, and determine whether they are suitable for use in microwave devices. Main performance (4π*M_s_*) is related to the loss, bandwidth, and power capacity of microwave devices. According to previous research [15], the change trend in Δ*H* also can be explained by the following equation:

(6) $$∆H=∆H_{int}+2.07\left(\frac{H_{a}^{2}}{4\pi M_{s}}\right)+1.5\left(4\pi M_{s}\right)P$$

where Δ*H_int_* is the intrinsic line widths, *H_a_* is the random anisotropy field, and the last part is attributed to the porosity of the grains. Thus, the reduction in Δ*H* was due to an enhancement of 4π*M_s_* and an increase in porosity. The results imply that the reduction in small grains can enhance the uniformity of ferrite grains and lower Δ*H* via appropriate CuO substitution [19,20]. The Δ*H* is a macroscopic physical quantity that reflects the damping experienced by the magnetization during its precession. It is related to the forward loss and working bandwidth of the device. The Δ*H* should be as narrow as possible.

## 4. Conclusions

In this work, Ni_0.22_Cu_0.31_Zn_0.47_Fe_2_O_4_ samples were synthesized via the solid-state preparation process, and the influence of Bi_2_O_3_–CuO flux addition on the microstructure, soft magnetic properties, and gyromagnetic properties of the obtained ferrites was studied. The XRD patterns showed that Bi_2_O_3_–CuO doping did not modify the spinel structure of the ferrites. The SEM images, as well as the density and grain size distribution of the samples, showed that with an appropriate Bi_2_O_3_–CuO content, the microscopic grains could fully grow under LTCC sintering and were more uniform and denser. Changes in the composition and microscopic properties induced corresponding changes in the soft magnetic properties and gyromagnetic properties, including *ε*′, *μ*′, *M_s_*, 4π*M_s_*, and Δ*H*. We obtained low-temperature-sintered NiCuZn ferrites by adding Bi_2_O_3_–CuO flux, and the soft magnetic properties and gyromagnetic properties of the doped ferrites could be tuned by changing the CuO content. These NiCuZn ferrites are promising for developing RF frequency multilayer devices, FRID fields, NFC systems, and chip inductors that are resistant to DC bias, where they can shield interference and enhance the working distance.

## Figures and Tables

**Table 1:** Theoretical density, bulk density, and porosity of the NiCuZn samples with 0.3 wt% Bi_2_O_3_ + *x*CuO addition (*x* = 0.2, 0.4, 0.6, and 0.8 wt%).

*x* Contents	Theoretical Density (g/cm^3^)	Bulk Density (g/cm^3^)	Porosity
0.2	5.32	4.43	16.7%
0.4	5.41	4.62	14.6%
0.6	5.48	4.88	10.9%
0.8	5.63	4.95	12.0%

**Table 2:** Lorentz fitting parameters of the NiCuZn samples with 0.3 wt% Bi_2_O_3_ + *x*CuO addition (*x* = 0.2, 0.4, 0.6, and 0.8 wt%).

Lorenz Fitting Parameters	*x* = 0.2 wt%	*x* = 0.4 wt%	*x* = 0.6 wt%	*x* = 0.8 wt%
*y*_0_	−32.02	−32.49	−32.34	−32.53
*x*_c_	3275.73	3318.48	3348.89	3321.99
*w*	372.89	331.99	242.85	251.21
*A*	−6398.64	−6485.31	−5106.76	−5075.78
*H*	−10.92	−12.44	−13.39	−12.86
Reduced chi-square	0.08	0.13	0.09	0.073
*R* ^2^	0.99	0.98	0.99	0.99

## Data Availability

Data is contained within the article.
